# P-683. What if Adult RSV Vaccine Uptake in the US was High Like in Scotland? Missed Opportunities to Reduce Public Health and Economic Burden of RSV

**DOI:** 10.1093/ofid/ofaf695.896

**Published:** 2026-01-11

**Authors:** Reiko Sato, Erica Chilson, Erin Quinn, Ahuva Averin

**Affiliations:** Pfizer, Inc., Collegeville, PA; Pfizer, 500 Arcola Road, Pennsylvania; Avalere Health, Boston, Massachusetts; Avalere Health, Boston, Massachusetts

## Abstract

**Background:**

Since June 2024, the United States (US) Advisory Committee on Immunization Practices has recommended routine vaccination against respiratory syncytial virus (RSV) for all adults aged ≥ 75 years. Despite the recommendation, uptake of RSV vaccine among those aged ≥ 75 years remains low, especially compared with other countries that have similar recommendations. We therefore evaluated the potential public health and economic impact of improving vaccine coverage among US adults aged ≥ 75 years, based on observed uptake within the same age group in Scotland.

Public health outcomes associated with alternative vaccination strategies for US adults aged ≥75 years

**Figure:**
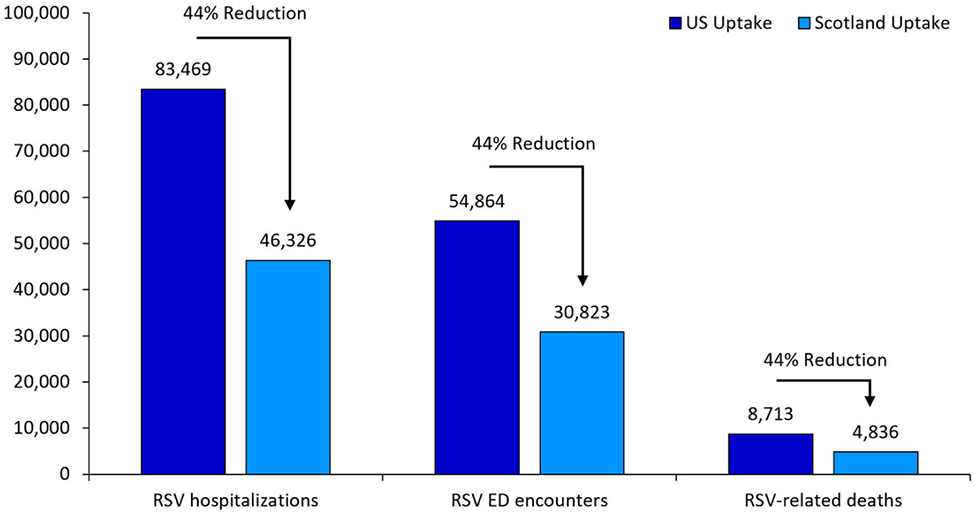

**Methods:**

We developed a cohort model to depict public health and economic outcomes associated with RSV and the single-season impact of routine immunization with RSV vaccines available during the first season. Severe public health outcomes included RSV episodes requiring hospitalizations (RSV-H) or emergency department (ED; RSV-ED) care, respectively, and RSV hospitalization-associated deaths; economic outcomes included costs associated with medical care for RSV. Vaccine effectiveness (vs. RSV-H: 79%, vs. RSV-ED: 78%) was based on real-world observational data from the 2023-24 RSV season in the US. Two different uptake scenarios were considered: Scenario 1 (23.6%, based on US uptake) and Scenario 2 (68.6%, based on uptake in Scotland).

**Results:**

With Scenario 1 (23.6% uptake), there were 83,469 RSV-H episodes, 54,864 RSV-ED episodes, and 8,713 RSV-related deaths; associated medical care costs totaled $2.3 billion. With Scenario 2 (68.6% uptake), there were 46,326 RSV-H episodes, 30,823 RSV-ED episodes, and 4,836 RSV-related deaths; associated medical care costs totaled $1.3 billion, representing 44% reductions in all public health and economic outcomes in a single season.

**Conclusion:**

Findings suggest that if uptake of RSV vaccine among US adults aged ≥ 75 years was comparable to uptake in Scotland, nearly 40,000 additional hospitalizations, 25,000 additional RSV-ED visits, and 4,000 additional RSV-related deaths could have been prevented in the first season following vaccine administration. The US should improve targeted efforts to reach remaining unvaccinated eligible adults to prevent severe outcomes.

**Disclosures:**

Reiko Sato, PhD, Pfizer Inc: Stocks/Bonds (Public Company) Erica Chilson, PharmD, Pfizer Inc: Stocks/Bonds (Public Company) Erin Quinn, BS, Pfizer Inc: Advisor/Consultant Ahuva Averin, MPP, Pfizer Inc: Grant/Research Support

